# Food Safety Distribution Systems Using Private Blockchain: Ensuring Traceability and Data Integrity Verification

**DOI:** 10.3390/foods14081405

**Published:** 2025-04-18

**Authors:** Seung Eel Oh, Jong-Hoon Kim, Ji-Young Kim, Jae-Hwan Ahn

**Affiliations:** Food Safety and Distribution Research Group, Korea Food Research Institute, Wanju 55365, Republic of Korea; jhkim@kfri.re.kr (J.-H.K.); jykim@kfri.re.kr (J.-Y.K.); jhahn@kfri.re.kr (J.-H.A.)

**Keywords:** food safety, blockchain technology, food supply chain, traceability system, data integrity, IoT-based monitoring, smart food distribution

## Abstract

The complexity of contemporary supply chains and the rise in foodborne illness cases have made ensuring food safety and traceability a top responsibility on a worldwide scale. Traditional traceability systems are prone to data tampering, fragmentation, and limited compatibility. Public blockchains have scalability, latency, and privacy problems that limit their use in real-time food safety systems, despite the fact that blockchain provides a secure data structure. Using Hyperledger Fabric, GS1 EPCIS standards, and Internet of Things-enabled environmental sensors, this paper suggests a private blockchain-based food safety monitoring system. To guarantee fault-tolerant, high-throughput processing in a permissioned blockchain setting, a Raft consensus mechanism was used. Hyperledger Caliper was used to benchmark the system once it was deployed with four nodes. According to experimental data, transaction throughput peaked at 230.2 TPS and averaged 207.4 ± 10.2 TPS. As the network grew from two to four nodes, latency increased somewhat from 259.3 ± 9.5 ms to 278.7 ± 9.1 ms, while block finalization time stayed below 3.184 ± 0.113 s. Over 114,925 documented transactions, data integrity was confirmed to be flawless. These results demonstrate that private blockchain technology can provide effective, scalable, and impenetrable food traceability, boosting openness and confidence throughout food networks.

## 1. Introduction

Since foodborne illnesses continue to provide serious health risks and financial burdens, ensuring food safety and transparency in supply chains has emerged as a crucial global concern. The World Health Organization (WHO) estimates that, each year, 600 million cases of foodborne diseases and about 420,000 fatalities are caused by contaminated food [1]. In addition to endangering the public’s health, contaminated food products severely harm industries’ bottom lines through recalls, fines from the government, and harm to their brand [2]. These dangers are increased by the complexity of contemporary food supply chains, which involve numerous middlemen in various geographical locations, making it challenging to promptly locate the sources of contamination and ensure regulatory compliance [3,4].

Food traceability systems have been extensively utilized to oversee the progression of food products from manufacturing to consumption, with the objective of addressing the prevailing issues [5,6]. Conventional traceability systems are predicated on manual record-keeping and centralized databases, which are often characterized by fragmentation, a predisposition to errors, and susceptibility to manipulation [7]. Moreover, these systems lack interoperability among a multitude of stakeholders, resulting in ineffective information exchange and challenges with real-time monitoring [8,9]. There is an urgent need for a more transparent, dependable, and automated traceability system that guarantees data integrity and stops fraudulent actions in the food distribution chain due to growing global regulatory demands for stricter food safety regulations [10,11].

Because of its ability to enable safe, decentralized, and impenetrable data management in supply chains, blockchain technology has been heralded as a revolutionary answer to the aforementioned problems [12]. Blockchain technology, as opposed to conventional, centralized systems, enables a wide range of stakeholders, including manufacturers, distributors, retailers, and regulatory bodies, to access and validate shared transaction records without the need for middlemen [13]. It has been demonstrated that the immutability, transparency, and real-time traceability of the system greatly improve food safety monitoring and shorten the time needed to identify and address contamination situations [14,15]. Numerous studies have shown how well blockchain works to increase consumer trust, ensure product authenticity, and improve supply chain visibility [16,17]. Nevertheless, a large number of current blockchain-based food safety solutions depend on public blockchain networks, which have a number of drawbacks, such as expensive transaction fees, scalability issues, and privacy issues with data [18]. Because they employ energy-intensive consensus techniques (such as Proof of Work), public blockchain systems like Ethereum and Bitcoin are less appropriate for real-time food traceability applications that demand quick transactions and economical operation [19].

This paper suggests a Hyperledger Fabric-based private blockchain-based food safety distribution system to overcome these drawbacks. The suggested solution makes use of a Raft consensus algorithm for optimal transaction validation, IoT sensors for real-time environmental monitoring, and GS1 EPCIS standards for food traceability. Compared to conventional public blockchain systems, this method offers increased scalability, better transaction efficiency, and improved data privacy by putting in place a permissioned blockchain network [20]. Additionally, performance tests were carried out to evaluate the system’s general scalability, data correctness, and transaction throughput in actual food delivery scenarios.

By proving that a scalable, effective, and privacy-preserving food traceability system is feasible, this study advances blockchain-based food safety solutions. The suggested system guarantees data integrity, real-time monitoring, and quick response capabilities in food supply chains by combining blockchain technology with Internet of Things-based environmental monitoring. For supply chain managers, food safety authorities, and technology developers looking to improve the efficiency and transparency of food delivery systems, these findings offer insightful information.

## 2. Materials and Methods

The proposed private blockchain-based food safety distribution system integrates Hyperledger Fabric, IoT-based real-time monitoring, and GS1 EPCIS traceability standards to enhance food traceability and data integrity [20]. This section provides details on the system architecture, blockchain network configuration, and evaluation methods to ensure reproducibility. The system was designed to support secure, immutable, and real-time traceability of food distribution records while maintaining scalability and efficiency.

### 2.1. System Architecture and Design

The system comprises three primary components: an IoT-enabled data acquisition layer, a private blockchain network, and a smart contract-based processing module. These components work in coordination to ensure seamless data collection, secure storage, and efficient traceability in food distribution systems. The system architecture ensures real-time traceability, data consistency across multiple stakeholders, and a decentralized yet secure platform for food safety monitoring.

#### 2.1.1. IoT-Enabled Data Acquisition Layer

**Figure 1:**
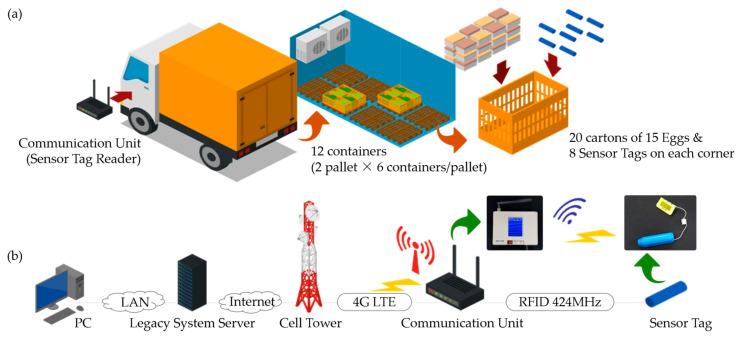
IoT-based food safety monitoring system: (**a**) device and sample configuration, (**b**) data flow and communication network.

In order to ensure continuous monitoring of food safety conditions during distribution, temperature and humidity sensor tags (ST.2018.lte.a02; ASN Inc., Anyang, Republic of Korea) were deployed inside food containers. These compact sensor tags, which were approximately the size of a finger, were placed directly alongside the food items in order to capture real-time environmental data. The sensor tags recorded temperature and humidity readings at five-minute intervals and transmitted the data via RFID (424 MHz) to a Communication Unit (CU.2018.lte.a02; ASN Inc., Republic of Korea) installed within the transport vehicle. The configuration of the sensor placement and food sample is depicted in Figure 1a.

The Communication Unit (CU), equipped with both an LTE communication modem and a GPS module, handled data transmission to the food safety distribution legacy system. The overall data flow and communication network are illustrated in Figure 1b. The data collection and transmission process was structured as follows:

- Sensor tags transmitted temperature and humidity data to the CU via RFID (424 MHz) every 5 min.
- The CU transmitted both sensor data and GPS location data to the food safety distribution legacy system via LTE every 10 min.
- GPS location data were recorded every 3 min and stored temporarily before being transmitted along with the environmental data.
- The collected data could be accessed in real time via the web-based monitoring platform, requiring administrator login credentials.
- In the event of a temporary communication failure or transport accident, the Communication Unit is designed to retain sensor data in its local memory and resume transmission once connectivity is restored. This fault-tolerant buffering mechanism ensures that no data are lost, even in emergency or unexpected conditions, thereby preserving complete traceability records.

For this experiment, the sensor tags were placed inside ventilated plastic containers, commonly used for refrigerated food transportation (Figure 1a). Each container measured approximately 500 mm (L) × 350 mm (W) × 300 mm (H), was made of PE material, and featured perforations for airflow and drainage. Each container held 20 cartons of 15 eggs as food samples, with eight sensor tags strategically placed at the eight corners—four at the bottom and four at the top—to ensure comprehensive environmental monitoring.

A total of 12 containers were prepared and loaded into a refrigerated transport vehicle for distribution. The Communication Unit (CU) was installed next to the driver’s seat, ensuring that it could efficiently receive RF signals from sensor tags placed inside the refrigeration compartment. This configuration allowed for uninterrupted data collection and transmission throughout the transportation process.

The overall device setup and communication architecture are illustrated in Figure 1, which provides a detailed schematic representation of sensor placement (Figure 1a), CU positioning, and the data flow between devices and the food safety distribution legacy system (Figure 1b).

#### 2.1.2. Private Blockchain Network

**Figure 2:**
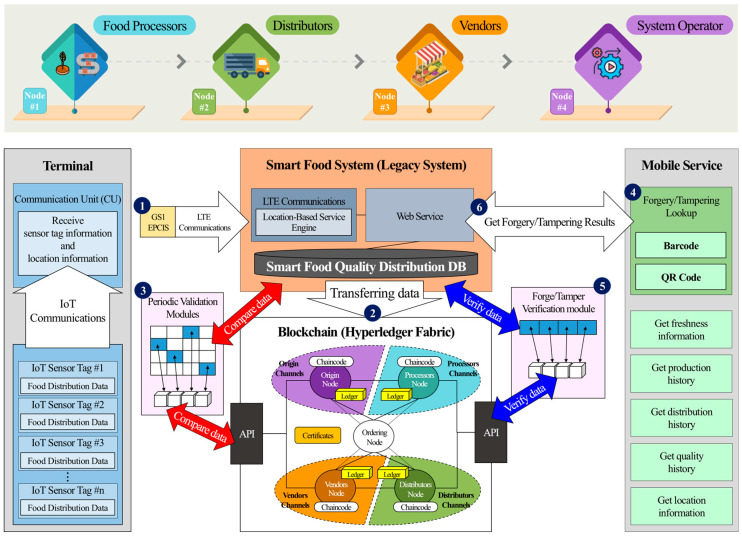
Blockchain-integrated smart food safety system: data flow and tamper verification process.

To ensure a secure, transparent, and verifiable food safety monitoring system, a private blockchain network was implemented using Bowledger, a Hyperledger Fabric-based framework. This network operates as a permissioned blockchain, where only authorized entities—including food processors, distributors, vendors, and the system operator—can participate in transaction validation and block verification. The architecture of this blockchain-integrated food safety distribution system is illustrated in Figure 2.

As shown in Figure 2, the proposed blockchain network is composed of four peer nodes, each representing a critical entity in the food distribution chain. The numbering of these nodes is arbitrary and follows the general food distribution cycle for clarity:

- Node 1: food processors;
- Node 2: distributors;
- Node 3: vendors;
- Node 4: system operator (blockchain administrator).

The blockchain network integrates GS1 EPCIS (Electronic Product Code Information Services) standards, ensuring global interoperability in food traceability by structuring event-based records that capture key attributes such as product ID, location, timestamp, and event type [21].

The system architecture follows a structured process to ensure traceability, tamper resistance, and real-time verification of food safety data. The six key processes depicted in Figure 2 are as follows:

1. IoT-Enabled Data Acquisition:
  Temperature and humidity sensor tags (ST.2018.lte.a02; ASN Inc., Republic of Korea) continuously collect environmental data from food containers. These sensor tags transmit data to a Communication Unit (CU.2018.lte.a02; ASN Inc., Republic of Korea) via RFID (424 MHz). The CU consolidates sensor readings and forwards the collected information—along with GPS location data—to the Smart Food System via LTE communication.
2. Blockchain Data Storage:
  The Smart Food System processes the received data, converts them into JSON format, and then securely transmits them to the blockchain network via an API. This ensures that food distribution records remain immutable and verifiable throughout the supply chain.
3. Data Integrity Validation:
  To prevent unauthorized modifications, the blockchain periodically cross-verifies stored transactions against data maintained in the Smart Food System database. A checksum-based validation mechanism is applied to detect discrepancies while minimizing computational overhead.
4. Tamper Detection via Mobile Interface:
  Consumers and supply chain managers can verify product authenticity by scanning QR codes or barcodes using a mobile application. The blockchain retrieves the corresponding transaction history and displays real-time food traceability data.
5. Forgery Detection Module:
  The system checks for data manipulation attempts by retrieving the transaction hash from the blockchain and comparing it against records stored in the Smart Food System. Any discrepancies indicate potential tampering.
6. Tamper Verification Response:
  If data integrity violations are detected, the Smart Food System generates an alert and notifies the requesting device with the verification results.

The food safety distribution legacy system not only collects temperature, humidity, and location data from the IoT-based data acquisition layer but also computes real-time food quality metrics. However, due to concerns over data scalability and efficient storage management, only 11 essential data fields are transmitted to the blockchain to ensure data integrity and tamper-proof recording, as shown in Table 1.

To maintain efficiency and scalability, blocks are generated every five minutes, ensuring consistent and structured transaction logging. The blockchain network was deployed on standard mid-range hardware, which provides sufficient computing power for processing transactions while remaining cost-effective and practical for real-world implementation. The system was implemented using open-source tools and a containerized architecture (Docker and Docker Compose), allowing modular deployment and portability. The blockchain nodes were orchestrated using CLI-based configurations and standard Hyperledger Fabric network scripts. For institutions with limited technical staff, the setup process may require initial support from IT professionals familiar with Docker, Linux environments, and Fabric network structures. However, routine operation—including transaction submission, monitoring, and ledger synchronization—can be conducted via an intuitive web-based dashboard and API endpoints, reducing the need for continuous blockchain-specific expertise. Each blockchain node operates with the following specifications:

- CPU: Intel^®^ Core i5-8400 @ 2.80 GHz (Intel Corporation, Santa Clara, CA, USA; standard desktop-class processor);
- RAM: 8 GB (DDR3 PC3-12800, Samsung Electronics Co., Ltd., Suwon, Republic of Korea; adequate for handling blockchain operations in a permissioned network);
- Storage: 465 GB HDD (Seagate BarraCuda ST3500413AS, Seagate Technology LLC, Fremont CA USA; sufficient for storing transaction logs and smart contract execution data);
- Operating system: Ubuntu 20.04 LTS (Canonical Ltd., London, UK; widely used, stable, and lightweight for blockchain environments).

The Raft consensus algorithm is utilized to enhance transaction efficiency and ensure high availability [22]. Unlike Byzantine Fault Tolerance (BFT)-based consensus mechanisms, Raft employs a leader-based approach, where Node 4 (system operator) serves as the leader responsible for coordinating transaction order and block generation [23,24]. The remaining nodes act as followers, replicating transactions and ensuring ledger consistency. This design optimizes consensus performance, minimizes latency, and provides a fault-tolerant environment.

**Figure 3:**
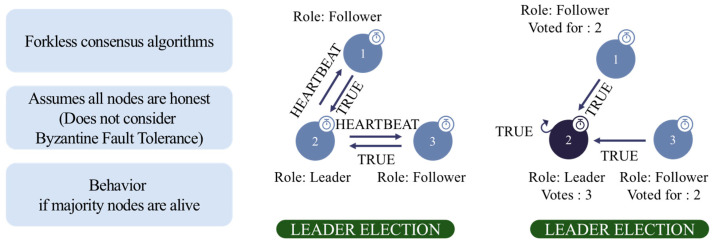
Raft consensus-based leader election and transaction validation in the blockchain network.

As illustrated in Figure 3, the Raft consensus mechanism follows a leader election-based mining system, ensuring that only one designated node is responsible for block creation at any given time [25]. The consensus mechanism operates under Crash Fault Tolerance (CFT), assuming that all nodes behave honestly and that the system remains operational as long as a majority of nodes are active. Unlike Byzantine Fault Tolerance (BFT) algorithms, this model does not account for malicious actors but instead prioritizes stability and efficiency in a controlled permissioned environment. To facilitate secure and seamless data exchange, the blockchain network uses the following dedicated ports:

- 6060/TCP: memory profiling for blockchain nodes;
- 8080/TCP: API-based transaction handling;
- 7845/TCP: gRPC remote procedure calls for node coordination;
- 7846/TCP: peer-to-peer (P2P) block synchronization.

By integrating IoT-driven monitoring, secure data storage, and blockchain-based verification, the proposed system establishes a tamper-resistant, transparent, and efficient food safety monitoring framework. This approach strengthens supply chain accountability, enhances regulatory compliance, and ensures consumer trust in food distribution data. To ensure secure remote access and protect against unauthorized intrusion, the system is implemented using a multi-layered security architecture. All blockchain peer nodes and communication endpoints are authenticated using a Certificate Authority (CA) under the Hyperledger Fabric framework. Transport Layer Security (TLS) is enforced for all data transmissions between nodes, preventing packet sniffing or man-in-the-middle attacks. Furthermore, the API gateway that interfaces between the blockchain and external applications is protected through access control lists, encrypted tokens, and rate limiting mechanisms to mitigate common attack vectors. The system is also deployed in a Docker-based container environment with restricted network access, minimizing the attack surface and allowing for isolated execution of each blockchain component. These security mechanisms collectively ensure that the proposed blockchain system remains resilient against external threats while allowing for authorized remote operation in distributed food safety monitoring scenarios.

#### 2.1.3. Smart Contract-Based Processing Module

**Figure 4:**
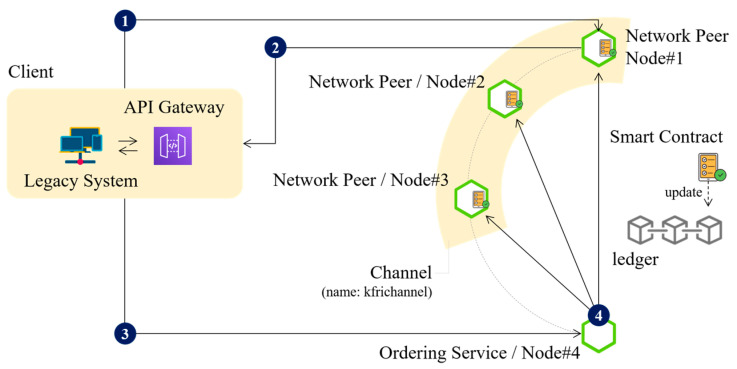
Smart contract execution workflow in the blockchain network.

The smart contract-based processing module was implemented to ensure automated validation, data integrity enforcement, and efficient transaction execution within the blockchain network. Smart contracts were deployed on the Hyperledger Fabric framework using Chaincode, enabling a permissioned environment where only authorized transactions were processed. The execution workflow of smart contracts follows a structured transaction lifecycle, as illustrated in Figure 4.

The smart contract execution workflow consists of four sequential processes:

1. Transaction Proposal Submission:
  A client retrieves relevant food safety data from the legacy system and submits a transaction proposal via the API gateway to the designated endorsement peer. The transaction is proposed to a predefined blockchain channel, “kfrichannel”, ensuring that only authorized network participants can process transactions.
2. Smart Contract Execution and Endorsement:
  Upon receiving the transaction proposal, each network peer verifies the client’s identity and authorization. If the provided credentials are valid, the assigned smart contract (Chaincode) is executed. Based on the execution results and the predefined endorsement policy, each peer signs a response indicating YES (approved) or NO (denied) and sends it back to the client.
3. Transaction Ordering and Submission:
  The client collects endorsement responses, assembles a transaction including read/write sets and endorsement metadata, and submits it to Node 4, which serves as the ordering service in the blockchain network.
4. Block Finalization and Ledger Update:
  The ordering service aggregates transactions, orders them into blocks, and distributes them to all network peers. If a transaction meets the defined validation criteria, the blockchain updates the state database and commits the transaction to the ledger.

This smart contract execution process ensures decentralized verification, immutability of records, and scalability of transaction handling, making it an integral part of the food safety blockchain network. By integrating smart contracts into the blockchain infrastructure, the system enhances automation, transparency, and security in food traceability and regulatory compliance [26]. To verify the correct execution of smart contracts and overall transaction lifecycle, a benchmarking framework, Hyperledger Caliper, was employed. This system component ensured that each smart contract invocation was tracked, evaluated, and recorded under preconfigured parameters during performance testing.

## 3. Results

This section presents the results obtained from the implementation of the private blockchain-based food safety monitoring system. The key findings focus on the system’s transaction performance, data integrity validation, and blockchain scalability and latency analysis. Specifically, Section 3.1, Section 3.2 and Section 3.3 evaluate transaction throughput, data integrity, and system scalability to assess the overall performance of the proposed blockchain system.

### 3.1. System Performance Evaluation

**Figure 5:**
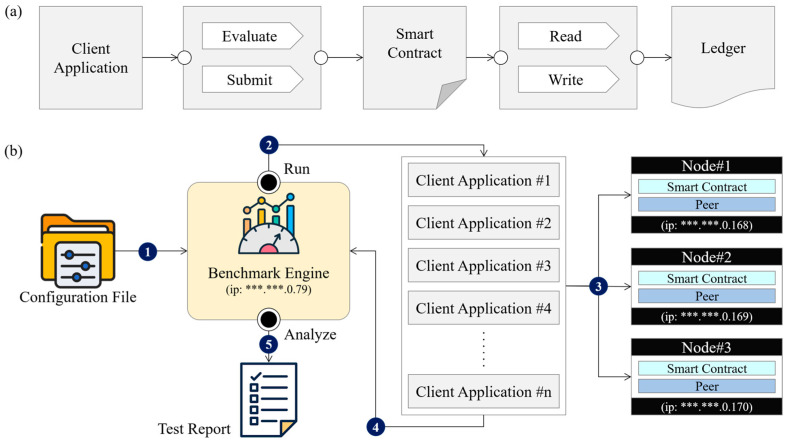
Measured transaction processing performance in the blockchain network: (**a**) benchmarking workflow, (**b**) performance evaluation process. Part of the IP address is asterisked due to security issues.

To evaluate the system’s transaction processing capabilities, benchmarking tests were conducted using Hyperledger Caliper, a widely adopted open-source blockchain performance evaluation framework [27]. The blockchain network was deployed in a controlled test environment consisting of three peer nodes and one ordering service node. This environment was implemented at a food distribution facility operated by the Korea Food Research Institute, where the blockchain system was tested in conjunction with a refrigerated transport vehicle equipped with IoT-enabled sensor units. The benchmarking process was repeated 10 times, and the results were averaged to ensure statistical reliability. Hyperledger Caliper served not only as a benchmarking tool but also as a verification system to assess whether the blockchain nodes and smart contract executions adhered to the expected operational parameters under varying network loads. The benchmarking workflow and transaction processing results are summarized in Figure 5.

Figure 5 illustrates the workflow used to measure transaction throughput in the blockchain network. The benchmarking process follows a structured five-step approach to ensure accurate performance evaluation:

1. Configuration file preparation: benchmark parameters, such as transaction volume, network size, and transaction rate, were specified and provided to the Benchmark Engine.
2. Client Application generation: the Benchmark Engine interpreted the configuration parameters and created multiple Client Applications, each responsible for submitting transactions to the blockchain network.
3. Transaction execution: Client Applications generated transactions and submitted them concurrently to the peer nodes in the network.
4. Performance data collection: each Client Application recorded transaction latency (read/write performance) and sent the results back to the Benchmark Engine.
5. Test report generation: the Benchmark Engine analyzed the collected data and produced a performance evaluation report, including average TPS, maximum TPS, and read/write transaction rates.

During the benchmarking process, max commit (maximum write speed) for 10,000 transactions was recorded as follows:

- Average TPS (mean ± standard deviation): 207.4 ± 10.2;
- Maximum TPS: 230.2.

**Figure 6:**
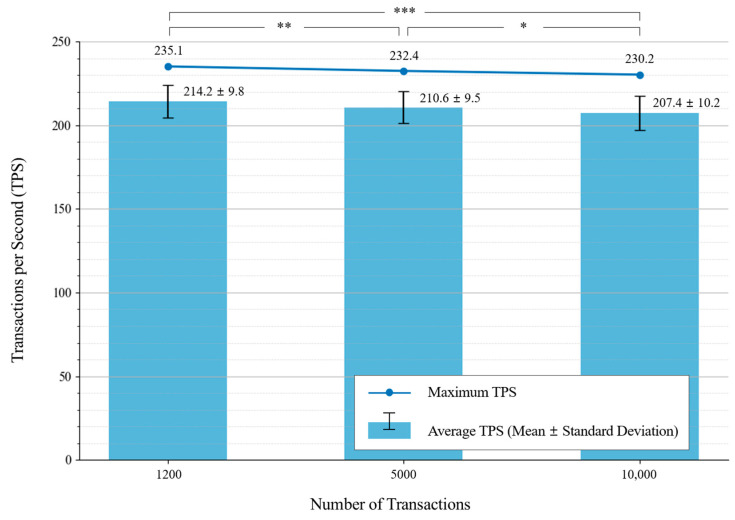
Blockchain transaction throughput under different network loads. Asterisks indicate statistically significant differences between groups: *p* < 0.05 (*), *p* < 0.01 (**), *p* < 0.001 (***).

The benchmarking tests were conducted under three different network load conditions, with transaction volumes of 1000, 5000, and 10,000 transactions. The results of average transaction throughput (TPS) under different network loads are presented in Table 2, and the corresponding graphical representation is shown in Figure 6.

To examine whether the slight decrease in transaction throughput was statistically significant, a pairwise significance test was performed between the three groups. The results showed statistically significant differences between all tested transaction volume conditions:

- Condition 1: 1000 vs. 5000 transactions: *p* = 0.0090 (*p* < 0.01, denoted as **);
- Condition 2: 5000 vs. 10,000 transactions: *p* = 0.0227 (*p* < 0.05, denoted as *);
- Condition 3: 1000 vs. 10,000 transactions: *p* = 0.0000 (*p* < 0.001, denoted as ***).

Figure 6 shows the benchmarked transaction processing throughput under varying transaction loads. The bar plot represents the mean ± standard deviation of transaction throughput, while the line plot indicates the maximum TPS recorded. Statistical significance between groups is denoted using asterisks: *p* < 0.05 (*), *p* < 0.01 (**), *p* < 0.001 (***) [28]. These statistical differences indicate that, although the numerical differences in TPS values appear small, they are statistically significant, meaning the system experiences a measurable decrease in throughput as transaction volume increases.

The results confirm that the proposed blockchain-based food safety monitoring system can handle high transaction loads with stable throughput. The slight decrease in TPS as transaction volume increases is expected due to the additional processing and network overhead required to manage larger transaction batches. However, the observed decrease is minimal, and even at the highest transaction volume (10,000 transactions), the system maintained a peak TPS of 230.2, demonstrating its scalability.

Furthermore, the statistically significant differences in transaction throughput indicate that, although the system remains efficient, higher transaction loads introduce a measurable impact on processing efficiency [29]. These results suggest that, while the system is well suited for real-time transaction processing in food safety monitoring, future optimizations could focus on further improving throughput stability under increased network demand.

### 3.2. Data Integrity Validation

To verify the integrity and consistency of food distribution records stored on the blockchain, data integrity validation tests were performed over a 50-day period, divided into ten intervals of five days each. The 50-day duration was selected based on the typical shelf life and distribution cycle of perishable food products, such as eggs and fresh produce. This allowed for a realistic evaluation of long-term data acquisition, sensor degradation, and transaction stability over time. The validation process involved comparing the original transaction data from the Smart Food System with the blockchain-stored JSON records across all peer nodes. The process was automated using a checksum-based verification method, ensuring that any discrepancies due to data corruption or unauthorized modifications could be detected [30].

**Figure 7:**
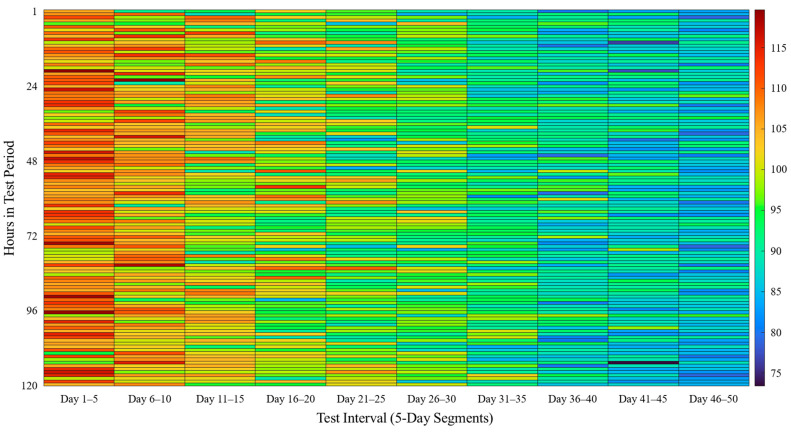
Heatmap of transactions logged per hour.

Figure 7 represents the hourly transaction logging patterns over 50 days using a heatmap, where red shades correspond to higher transaction volumes and blue shades indicate lower transaction volumes. The x-axis represents the test interval (in days), while the y-axis represents the time interval (in hours) across the 120 h span of each test period. The color intensity shows the number of transactions logged per hour.

The heatmap in Figure 7 reveals three key trends and their corresponding findings:

- Gradual Decline in Transaction Volume Over Time: The experiment involved food samples that were gradually removed over time, reducing the number of active sensors. Consequently, transaction counts declined over successive test intervals. In the Day 1–5 interval, transactions logged per hour ranged from 95 to 119, with a higher concentration of red hues. By the Day 45–50 interval, transaction counts had decreased to a range of 74 to 96, with more blue hues appearing.
- Fluctuations in Transaction Logging: Although an overall decreasing trend was expected due to sample removal, intermittent fluctuations in transaction logging were observed. These fluctuations were particularly prominent in the early stages of the experiment, as indicated by patches of sudden color shifts in the first 15 days.
- Impact of IoT Network Conditions on Data Transmission: The data transmission mechanism relied on IoT sensor tags communicating with Communication Units (CUs) over 4G LTE. When network conditions degraded (e.g., due to interference or weak signals), sensor data were temporarily buffered within the CU and transmitted later when the connection improved. This behavior explains the observed spikes and delays in transaction logging, particularly in the initial phases where data transmission was most intensive.

These findings suggest that data transmission fluctuations were primarily a result of IoT sensor and CU performance, rather than blockchain performance. The blockchain effectively recorded transactions once they were successfully transmitted, ensuring that no data were permanently lost, despite intermittent delays in logging.

Each transaction entry recorded on the blockchain was compared with the corresponding entry in the food safety distribution legacy system to confirm consistency. The checksum validation mechanism performed an exact comparison between the two datasets to detect discrepancies. Across ten repeated validation tests, zero discrepancies were found, confirming that all recorded transactions remained tamper-proof and consistent throughout the storage period. This result highlights the reliability of the blockchain in preventing unauthorized modifications and ensuring long-term data integrity.

Table 3 provides a summary of the number of transactions recorded during each five-day validation interval, along with the number of discrepancies detected and the overall integrity verification status. The following trends can be observed:

- Stable and Consistent Data Integrity: Over the course of 50 days, a total of 114,925 transactions were logged on the blockchain. No discrepancies were detected in any of the ten validation tests, confirming the blockchain’s ability to preserve data accuracy.
- Gradual Decrease in Transactions Logged Over Time: The number of transactions recorded per interval decreased progressively from 12,875 (Day 1–5) to 10,345 (Day 46–50). This decline corresponds to the reduction in active food samples and sensor tags, as food items were gradually removed for testing and evaluation.
- Consistency in Blockchain Record-Keeping: Despite the decrease in transaction volume, the blockchain system successfully recorded and verified all transactions, demonstrating its robustness in maintaining traceability and tamper-proof storage. The periodic validation process confirmed that the blockchain ledger remained fully synchronized across all participating nodes.

The validation results confirm that data recorded on the blockchain remained consistent with the original source data, demonstrating the system’s ability to ensure data integrity over an extended period of operation.

- No discrepancies were detected, meaning that all transactions were successfully validated without tampering.
- The blockchain network maintained data consistency across all peer nodes, ensuring synchronized records in the distributed ledger.
- The checksum-based verification method effectively detected any anomalies, providing a robust approach to securing food distribution records against unauthorized modifications.

While temporary transaction logging fluctuations were observed (as indicated in Figure 7), these did not affect the accuracy or integrity of the stored data. Instead, they reflect the IoT sensor and CU communication process and highlight the importance of optimizing wireless transmission in future deployments. Overall, these findings demonstrate that the blockchain network successfully preserved transaction accuracy, ensuring a tamper-resistant and fully traceable food safety monitoring system.

### 3.3. Blockchain Scalability and Latency Analysis

To evaluate the impact of network expansion on transaction processing, scalability and latency tests were conducted over ten repeated trials by varying the number of nodes in the blockchain network. The average transaction latency was measured as the time taken from transaction proposal to block confirmation. Similarly, block finalization time was analyzed to determine the effect of increasing peer nodes on network efficiency and consensus validation speed. In a blockchain network, the number of nodes plays a critical role in determining transaction latency, block finalization efficiency, and network resilience. While the minimum practical configuration for a permissioned blockchain network is typically three nodes, a two-node configuration was included in this study for experimental scalability and latency analysis, despite its inherent limitations. The performance implications of using only two nodes are outlined below:

- Consensus Constraints:
  The Raft consensus mechanism requires a majority (N/2 + 1) to reach consensus. In a two-node network, both nodes must remain operational at all times. If one node fails, the blockchain ceases to function due to an inability to finalize transactions.
- Lack of Redundancy and Security Risks:
  In a two-node setup, the failure of a single node leads to a complete network failure. Moreover, with only one validator, the system is more vulnerable to a single point of failure or potential malicious activity, which may compromise data integrity.
- Use Case Limitations:
  While two-node blockchains are unsuitable for full-scale production environments, they can be useful for controlled experimental conditions, isolated data exchange, and redundancy evaluation.

Despite these constraints, scalability and latency tests were conducted for blockchain networks with two, three, and four nodes to analyze performance trends as the network size increased. The results are summarized in Table 4.

**Figure 8:**
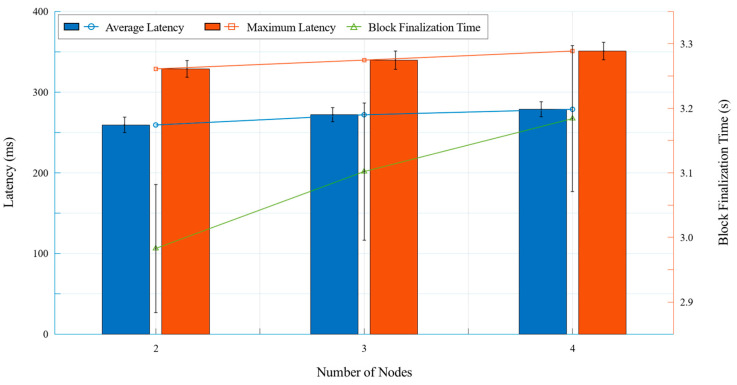
Blockchain scalability and latency analysis.

Figure 8 provides a graphical representation of the impact of network expansion on transaction latency and block finalization time. The x-axis represents the number of nodes, set at 2, 3, and 4, while the y-axis incorporates two distinct metrics. The left y-axis represents transaction latency in milliseconds, where average transaction latency is depicted by blue bars and a corresponding line, while maximum transaction latency is shown in orange. The right y-axis denotes block finalization time in seconds, illustrated by a green line. This visualization highlights the increasing trend in latency as the number of nodes grows, alongside the relatively stable progression of block finalization time, offering insights into the computational overhead introduced in a permissioned blockchain network utilizing the Raft consensus mechanism.

The latency and finalization time trends observed in Table 4 and Figure 8 indicate that, as additional nodes are introduced, transaction processing efficiency is moderately impacted but remains within an acceptable range for real-time food traceability applications.

- The average transaction latency increased from 259.3 ± 9.5 ms (two nodes) to 278.7 ± 9.1 ms (four nodes), indicating increased processing overhead in larger networks.
- The maximum latency ranged from 328.7 ± 10.2 ms to 350.8 ± 11.0 ms, reflecting the additional consensus processing time.
- The block finalization time exhibited a moderate increase from 2.983 ± 0.099 s to 3.184 ± 0.113 s, reflecting the additional consensus verification steps required for larger networks.

Despite the increasing latency, the four-node blockchain network maintained stable and efficient performance, ensuring transaction reliability while providing enhanced redundancy and fault tolerance. Furthermore, fault tolerance testing demonstrated that the blockchain remained operational even when a node was temporarily disconnected, verifying the resilience of the Raft consensus mechanism in ensuring continued transaction validation. Given these findings, the four-node configuration was determined to be the optimal upper limit for this study, as larger network sizes were beyond the intended scope. Future research should explore scalability beyond four nodes to examine long-term blockchain performance trends in large-scale deployments. While the network was configured with up to four nodes in this study, this reflects the minimum practical setup required to demonstrate performance under a permissioned blockchain architecture. A four-node configuration includes essential roles such as client, endorser, committer, and ordering service while preserving redundancy and consensus stability. Therefore, future studies should consider larger network topologies—such as configurations with six to eight nodes—in which each role can be represented by multiple nodes. This would allow for more comprehensive scalability testing under broader, real-world deployment scenarios.

## 4. Discussion

The findings of this study confirm that the integration of a private blockchain network with IoT-enabled food monitoring significantly enhances data integrity, transaction efficiency, and scalability in food safety management. The transaction performance evaluation using Hyperledger Caliper demonstrated that the system achieved an average transaction throughput of 207.4 ± 10.2 TPS, with a peak throughput of 230.2 TPS for 10,000 transactions. Figure 6 provides further insights into the system’s ability to maintain stable transaction processing even as the number of transactions increases. Although the transaction throughput exhibited a slight decline with higher transaction volumes, the statistical analysis confirmed that these differences were statistically significant. The *p*-values calculated for the throughput differences between transaction volumes (1000 vs. 5000, 5000 vs. 10,000, and 1000 vs. 10,000) indicated significant variations, highlighting the system’s sensitivity to increased network loads. This suggests that, while the blockchain network remains efficient, further optimization is necessary to sustain high performance under heavy workloads.

The data integrity validation tests further substantiate the system’s reliability in ensuring immutability and consistency in food safety records. Over a 50-day evaluation period, comprising 114,925 transactions across 10 validation cycles, no discrepancies were detected between the blockchain records and the food safety distribution legacy system. Figure 7 presents a heatmap visualization of transaction logging patterns over time, illustrating variations in transaction frequency. The results revealed that the number of recorded transactions per hour gradually declined as this study progressed, primarily due to the gradual reduction in available food samples over time. However, fluctuations in transaction logging rates were observed, particularly in earlier intervals, likely attributed to temporary communication disruptions or latency in sensor-to-blockchain data transmission. These fluctuations were effectively mitigated by the system’s fail-safe mechanisms, which ensured data recovery and seamless record synchronization. The findings confirm that the implemented blockchain model effectively prevents unauthorized modifications and ensures end-to-end traceability, addressing key challenges in conventional food safety tracking.

The scalability and latency analysis provided additional insights into the system’s performance trends. The system maintained low transaction latency and stable block finalization times across all tested configurations. As illustrated in Figure 8, while latency increased from 259.3 ± 9.5 ms (two nodes) to 278.7 ± 9.1 ms (four nodes) and block finalization time ranged from 2.983 ± 0.099 s to 3.184 ± 0.113 s, these values remained within acceptable thresholds for real-time food traceability applications. The visualization of these trends in Figure 8 further reinforces the observation that network expansion introduces only a moderate computational overhead, ensuring continued efficiency in transaction validation and record synchronization.

The inclusion of a two-node blockchain configuration in the analysis was particularly relevant for evaluating minimum network requirements for consensus and fault tolerance. While a two-node blockchain lacks redundancy and cannot tolerate node failures, its experimental inclusion provided a baseline performance reference for scalability testing. The results demonstrated that, even in a minimal configuration, transaction validation and ledger consistency were maintained, highlighting the efficiency of the Raft consensus mechanism. Furthermore, fault tolerance testing confirmed that the system remained operational despite the temporary disconnection of one node, reinforcing the resilience of Raft-based block ordering. This finding suggests that the blockchain system can recover from transient failures and continue processing transactions as long as the majority (N/2 + 1) of nodes remain functional. Although the blockchain network operates within a secured permissioned environment, it is susceptible to external threats if improperly configured. Therefore, the system incorporates authentication mechanisms, TLS encryption, and certificate authorities to prevent unauthorized access. In addition, remote access is managed through an API gateway that supports access control, limiting entry points for malicious actors. These security provisions ensure that the system remains protected against common cybersecurity threats while enabling controlled remote monitoring.

The findings of this study align with previous research on blockchain-based food traceability. Waghmare et al. (2024) and Xu et al. (2024) reported comparable transaction throughputs of 180–220 TPS in similar blockchain-enabled supply chain applications [7,14]. However, those studies primarily relied on Byzantine Fault Tolerance (BFT)-based consensus mechanisms, which require higher computational overhead and increased network latency due to multi-party validation processes. In contrast, this study suggests that Raft-based consensus provides a more efficient alternative for food traceability applications, particularly in environments where participant trust is pre-established. The results indicate that validation complexity can be reduced while maintaining network reliability, making Raft-based consensus a suitable choice for permissioned blockchain networks in food safety monitoring.

Despite the promising results, several limitations warrant further investigation. While the system exhibited high performance under controlled experimental conditions, its scalability in real-world deployments involving multiple supply chain entities, variable transaction loads, and cross-border regulatory compliance remains to be fully explored. Future studies should assess how increased network traffic and heterogeneous data integration affect blockchain efficiency in diverse supply chain environments.

While the Raft consensus mechanism is known to be more computationally efficient than Proof-of-Work (PoW)-based models, its energy consumption remains a crucial consideration for IoT-based applications where devices often operate under strict energy constraints. Recent studies, such as Paris and Long’s (2015) [31], have proposed methods to reduce Raft’s energy footprint—such as using smaller quorums and integrating “witness” nodes that participate in consensus with minimal storage and energy use. Although this study did not incorporate these optimizations, such approaches present promising directions for future enhancements. Therefore, future research should focus on evaluating and implementing energy-aware variants of Raft in the context of blockchain-enabled food safety monitoring.

The implemented smart contracts effectively automated compliance enforcement and data validation. However, their current structure remains limited to rule-based execution. Incorporating machine learning algorithms for anomaly detection and predictive food quality assessment could further enhance blockchain-based automation in food safety monitoring. Despite the adoption of GS1 EPCIS standards to enable data interoperability, the integration of blockchain systems with existing food safety and supply chain platforms—particularly ERP and regulatory compliance systems—remains an unresolved challenge. Legacy infrastructures typically rely on heterogeneous databases, proprietary data formats, and centralized control models that are not natively compatible with decentralized blockchain architectures. Addressing these disparities requires the use of middleware, API-based connectors, or data translation layers to facilitate secure and accurate information exchange. Future research should prioritize the development of lightweight interoperability frameworks that allow seamless synchronization of traceability data while maintaining compliance with industry standards and regulatory policies.

Finally, while this study primarily focused on supply chain stakeholders, consumer-facing applications could further strengthen public trust in food safety standards. The integration of QR-based verification mechanisms enabling consumers to access blockchain-stored product histories warrants further exploration. To support ease of deployment and practical adoption, the system was developed using the Hyperledger Fabric framework, which offers modular architecture, extensive community support, and documentation. The system can be deployed and maintained by IT personnel with moderate experience in Docker-based environments and blockchain frameworks, and it does not require advanced blockchain expertise for daily operations. For small- and medium-sized food safety organizations without internal technical teams, the initial setup may benefit from external support. However, the system’s user interface, API-based integration, and monitoring dashboard are designed for usability and long-term operability with minimal administrative overhead.

However, the effectiveness of such tools depends on consumer willingness to engage with the technology, their trust in the accuracy of the data, and ease of use in real-world scenarios. Factors such as interface accessibility, perceived transparency, and confidence in the neutrality of the platform can significantly influence adoption. Therefore, future research should incorporate interdisciplinary approaches that address human-centered design, behavioral psychology, and regulatory guidance to foster consumer trust and encourage widespread use of blockchain-enhanced food authentication systems.

## 5. Conclusions

This study proposed a private blockchain-based food safety monitoring system that integrates IoT-driven data acquisition, GS1 EPCIS traceability standards, and a Raft-based consensus mechanism to enhance traceability, data integrity, and transaction efficiency in food distribution networks. The research aimed to design a scalable permissioned blockchain architecture capable of real-time monitoring, tamper-proof record-keeping, and performance validation through empirical testing.

The system was evaluated through benchmarking, integrity validation, and scalability analysis. It achieved an average transaction throughput of 207.4 ± 10.2 TPS and a peak of 230.2 TPS under a 10,000-transaction workload. Statistical testing revealed significant differences across varying transaction volumes, highlighting the system’s sensitivity to load changes and ability to maintain high throughput. Over a 50-day validation period, a total of 114,925 transactions were recorded and verified with zero discrepancies, confirming the blockchain’s effectiveness in preserving data integrity. Additionally, scalability analysis demonstrated that average latency and block finalization time increased moderately from two to four nodes but remained within acceptable bounds for real-time applications.

The experimental results collectively demonstrate that the system met its design objectives. Specifically, it enabled immutable transaction logging, consistent data availability, and efficient blockchain performance under realistic network loads. These capabilities are critical for regulatory compliance and transparency in modern food supply chains.

Furthermore, the heatmap visualization of hourly transaction data highlighted the system’s resilience in handling real-world variations caused by sensor behavior and communication conditions. The Raft consensus mechanism maintained operational stability even with fluctuating transmission rates and temporary node disconnections.

While the system exhibited strong performance in a controlled testbed, further investigation is necessary for large-scale deployment scenarios. Future work should evaluate blockchain performance in cross-border, multi-stakeholder supply chains and extend scalability testing beyond four nodes. Additionally, this study did not evaluate the energy consumption of the Raft consensus algorithm, which is particularly relevant in IoT-based blockchain environments. Recent work by Paris and Long (2015) has proposed energy-efficient strategies such as quorum reconfiguration and the use of witness servers to minimize power usage while maintaining consensus integrity [31]. These methods are promising for low-power device deployment, such as Raspberry Pi-based nodes. Future studies should assess the applicability of these techniques to enhance the energy efficiency of Raft-based blockchain systems in food safety monitoring scenarios.

Interoperability with enterprise resource planning (ERP) systems and legacy traceability frameworks also remains a challenge. While GS1 EPCIS structuring enabled event-based data standardization, additional work is needed to ensure seamless integration across platforms. Incorporating machine learning models into smart contracts could support predictive food quality analysis and anomaly detection, enhancing automation and decision making. Lastly, consumer trust could be further strengthened by enabling QR-based verification for direct access to blockchain-stored food history, which may increase transparency and accountability.

In conclusion, this study demonstrates that private blockchain technology—when combined with IoT monitoring and smart contract automation—offers a viable, secure, and scalable solution for food traceability. The proposed system contributes to the development of intelligent food safety frameworks and lays the foundation for future improvements in energy optimization, system interoperability, and large-scale deployment strategies.

## Figures and Tables

**Table 1:** Essential data fields transmitted to the blockchain for food safety traceability.

Field Name	Variable	Data Type	Example Value	Description
Tag Sequence Number	TAG_SEQ	VARCHAR (20)	“200225011988”	Unique sensor tag identifier preventing reuse conflicts.
Trace Date	TRACE_DATE	TIMESTAMP (6)	20240210153045	Timestamp when the event was recorded (YYYYMMDDHHMMSS).
Event Code	EVENT_CODE	VARCHAR (10)	“05”	Code indicating the event type (e.g., shipment, anomaly detection).
Communication Unit ID	CU_ID	VARCHAR (20)	“41505052”	Unique ID of the Communication Unit transmitting the data.
Tag Humidity	TAG_HM	NUMBER (6,3)	22.300	Humidity recorded by the sensor (percentage).
Tag Temperature	TAG_TP	NUMBER (6,3)	21.840	Temperature measured by the sensor (°C).
Quality Value	QL_VAL	NUMBER	59.422916	Computed real-time food quality score.
Longitude	COORD_X	NUMBER	127.103256	GPS longitude coordinate of the recorded data.
Latitude	COORD_Y	NUMBER	37.546474	GPS latitude coordinate of the recorded data.
Quality Score	QL_Q	NUMBER	59.422916	Additional quality assessment metric.
Registration Date	REG_DATE	TIMESTAMP (6)	20240210153210	Timestamp of when data were registered in the system (YYYYMMDDHHMMSS).

**Table 2:** Average transaction throughput (TPS) under different network loads.

Number of Transactions	Average TPS (Mean ± Std Dev)	Maximum TPS
1000	214.2 ± 9.8	235.1
5000	210.6 ± 9.5	232.4
10,000	207.4 ± 10.2	230.2

**Table 3:** Data integrity validation results from ten repeated tests.

Test Interval	Transactions Logged	Discrepancies Detected	Integrity Status
Day 1–5	12,875	0	Verified
Day 6–10	12,489	0	Verified
Day 11–15	12,145	0	Verified
Day 16–20	11,802	0	Verified
Day 21–25	11,567	0	Verified
Day 26–30	11,289	0	Verified
Day 31–35	11,034	0	Verified
Day 36–40	10,812	0	Verified
Day 41–45	10,567	0	Verified
Day 46–50	10,345	0	Verified
Total	114,925	0	Verified

**Table 4:** Blockchain scalability and latency analysis (mean ± standard deviation).

Number of Nodes	Average Latency (ms)	Maximum Latency (ms)	Block Finalization Time (s)
2	259.3 ± 9.5	328.7 ± 10.2	2.983 ± 0.099
3	271.9 ± 8.8	339.6 ± 11.4	3.102 ± 0.106
4	278.7 ± 9.1	350.8 ± 11.0	3.184 ± 0.113

## Data Availability

The original contributions presented in this study are included in this article; further inquiries can be directed to the corresponding authors.
